# From palliative to curative treatment - stage IV mucinous adenocarcinoma, successfully treated with metronomic capecitabine in combination with Bevacizumab and surgery- a case report

**DOI:** 10.1186/s12885-015-1908-3

**Published:** 2015-11-10

**Authors:** Karolina Vernmark, Maria Albertsson, Bergthor Björnsson, Thomas Gasslander, Per Sandström, Xiao-Feng Sun, Annika Holmqvist

**Affiliations:** 1 Department of Clinical and Experimental Medicine, Linköping University, Linköping, Sweden; 2 Department of Oncology, Linköping University, S-58185 Linköping, Sweden; 3 Departement of Surgery, Linköping University, Linköping, Sweden

**Keywords:** Mucinous adenocarcinoma, Bevacizumab, Metronomic capecitabine

## Abstract

**Background:**

Mucinous adenocarcinoma (MAC) represents 6-19 % of all colorectal carcinoma. It is associated with poorer response to chemotherapy and chemoradiotherapy.

**Case presentation:**

A 27-year-old Swedish woman presented with stomach pain and weight loss, and was diagnosed with locally advanced MAC in the transverse colon as well as 3 liver metastases. Neoadjuvant treatment with fluorouracil, folinic acid and oxaliplatin (FLOX) failed due to several infections, pulmonary embolism and deteriorated performance status. The patient was therefore considered palliative. Palliative treatment with metronomic capecitabine 500 mg × 2 daily and bevacizumab every other week were initiated. After 4 months of treatment the tumors had regressed and the patient was able to undergo radical surgery, thereby changing the treatment intention from palliative to curative. No adjuvant chemotherapy was given. There were no signs of recurrence 9 months later.

**Conclusions:**

The role of the combination of metronomic capecitabine and bevacizumab in patients with MAC merits further investigation.

## Background

Mucinous adenocarcinoma (MAC) represents about 6–19 % of colorectal carcinomas (CRC) [1]. The WHO defines MAC as an adenocarcinoma in which at least 50% of the cancer tissue is composed of mucin [2].

Numerous studies have shown conflicting results regarding the prognosis of MAC compared to the more common non-mucinous adenocarcinoma (NMAC) [2, 3]. It has however been shown that MAC is less likely to be resected with negative surgical margins [4], more often metastasizes to lymph nodes [5, 6] and generally presents at a later stage compared to NMAC [2]. MAC is also more prone to local recurrence [4, 7] as well as peritoneal carcinomatosis [6, 8]. Although the characteristics of MAC have not been fully clarified, due to the low incidence of this type of tumor, studies have shown that MAC shows less p53 and p21 expression and less APC mutations compared to NMAC [2]. There also appears to be an increased frequency in BRAF mutation [1] and increased microsatellite instability (MSI) [9]. In our clinic it is not praxis to analyze these markers because the results would not affect our choice of treatment.

Compared to NMAC, MAC is associated with a poorer response to chemotherapy and chemoradiotherapy [10, 11], resulting in some restrictiveness to treatment. Here, we present a case of successful preoperative treatment and surgery of a patient with stage IV MAC.

## Case presentation

A 27-year-old Swedish woman with no family history of cancer presented with stomach pain, rectal bleeding and weight loss. A computed tomography (CT) revealed an 80 × 55 mm tumor in the transversal colon. Colonoscopy showed a stricturating, voluminous tumor with irregular polyps. Most of the tumor surface was covered with white necrotic tissue. Multiple biopsies from the site showed suspected adenocarcinoma, but the result was not conclusive. A biopsy was then taken from the abdominal wall, and MAC with K-ras mutation in codon 12, gene pGly 12ASP (c.35G > A) was found.

**Fig. 1:**
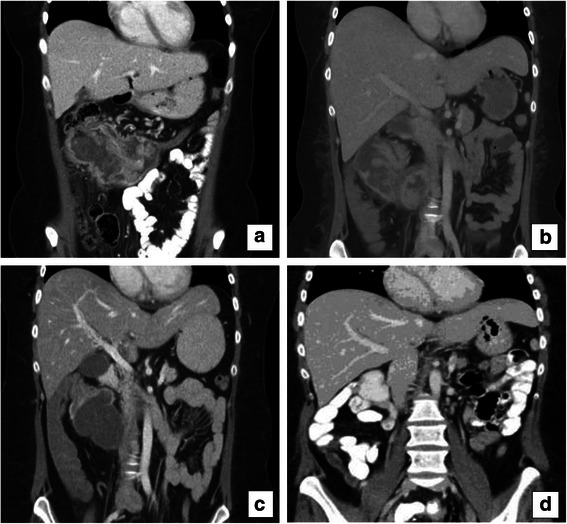
A computed tomography (CT) with contrast before treatment (**a**), 2 months after FLOX treatment (**b**), 4 months after treatment with metronomic capecitabine/bevacizumab (**c**) and 9 months after surgery (**d**)

Two months after the prior CT, a new CT showed that the tumor had grown rapidly, measuring 150 × 90 mm (Fig. 1a). It was locally advanced, seemed to infiltrate the duodenum and the mesentery, and involved the abdominal wall. Moreover, 3 small liver metastases were discovered, sized 12 mm, 7 mm and 4 mm, respectively. Carcinoembryotic antigen (CEA) was <5 μg/L and cancer antigen 19–9 (CA 19–9) was 27 kE/L, both within the normal range.

**Fig. 2:**
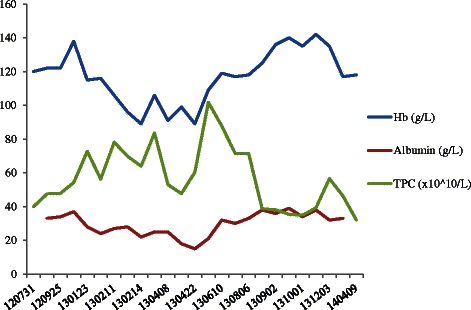
Platelet concentration (TPC), hemoglobin (Hb) and albumin levels over time during treatment

The primary tumor was originally considered non-resectable at multidisciplinary conference (MDC) and chemotherapy with neoadjuvant intention was initiated. The patient received 3 cycles of fluorouracil and oxaliplatin (FLOX), but unfortunately suffered from several infections and the dose was gradually reduced (Table 1). Even so, she rapidly deteriorated. She lost weight, slept most of the day and was dependent on parenteral nutrition (Table 1). She had anemia, thrombocytosis and hypoalbuminemia (Fig. 2), and also suffered from a pulmonary embolism. A CT scan showed similar tumor size compared to the previous CT (Fig. 1b).

**Table 1:** Patient’s BMI, chemotherapy doses given and performance status

Year	Month	BMI	Treatment	Dose	Performance status
2011		31.2			
2013	Feb	29.4	FLOX C1	Oxaliplatin: 150 mg Fluorouracil: 950 mg Calciumfolinat: 110 mg	0
	March	28.7	FLOX C2	Oxaliplatin: 140 mg Fluorouracil: 900 mg Calciumfolinat: 100 mg	1
	April	27.8	FLOX C3	Oxaliplatin: 120 mg Fluorouracil: 720 mg Calciumfolinat: 100 mg	1
	June	29.2	Capecitabine	500mgx1	1-2
	July		Capecitabine/ Bevacizumab C1	500 mg × 2/ 350 mg	1-2
	Aug		Capecitabine/ Bevacizumab C2	500 mg × 2/ 350 mg	0-1
			Capecitabine/ Bevacizumab C3	500 mg × 2/ 350 mg	
	Sept		Capecitabine/ Bevacizumab C4	500 mg × 2/ 350 mg	
			Capecitabine/ Bevacizumab C5	500 mg × 2/ 350 mg	
	Oct	29.8	Capecitabine/ Bevacizumab C6	500 mg × 2/ 350 mg	0-1
			Capecitabine/ Bevacizumab C7	500 mg × 2/ 350 mg	
2014	Jan	31.2			0

**Fig. 3:**
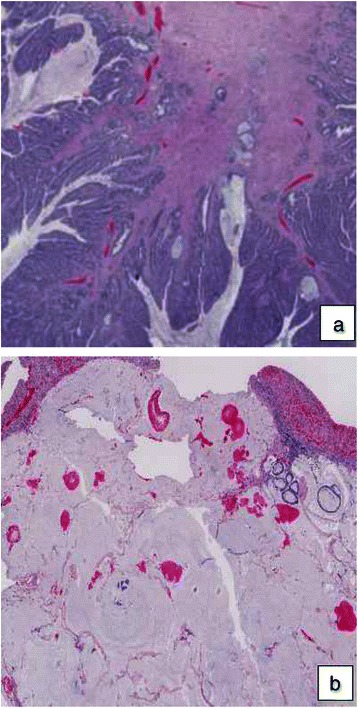
Histologic hematoxylin and eosin stained paraffin section of the patient’s tumor in the colon (**a**) and in the liver (**b**), respectively

The therapy was now focused on palliation and she was referred to palliative care. The treatment was switched to low dose metronomic (LDM) capecitabine 500 mg × 2 (500 mg twice daily without interruption) and bevacizumab 350 mg every other week. Within a short time she began to feel better, being able to stay up most of the day. After 2 months she described herself as feeling fresh and alert, and had gotten her appetite back. Her blood tests were restored to normal. After 3 months she was no longer in need of parenteral nutrition. She did not report any side effects of the given treatment. After 4 months a CT showed some regression in tumor size but clearly less solid component and low attenuating areas, indicating necrosis (Fig. 1c). At a new MDC the tumor was deemed resectable. Four and a half months after she began treatment with capecitabine/bevacizumab, she had surgery with right hemicolectomy, mesentery resection and omentectomy. Parts of the right rectus abdominis muscle were removed along with the tumor overgrowth. In the same operation, 3 of the liver metastases in segments 1, 3 and 4b were also removed. Yet another liver metastasis in segment 8 was identified with intraoperative contrast-enhanced ultrasound. It measured 5–6 mm and was treated with intraoperative radiofrequency treatment. Histopathological examination confirmed the diagnosis of a moderately differentiated MAC in the colon, measuring 90 × 60 mm. (Fig. 3) In addition, there was an 80x80 mm portion of the tumor that engaged the abdominal wall. There were large areas of necrosis in the tumor. Perineural growth was seen but no lymphovascular invasion. None of the 34 surgically removed lymph nodes contained metastasis. In the liver, 3 metastases of MAC were found, measuring 15 × 18 mm, 6 × 15 × 8 mm and 18 × 15 × 11 mm, respectively. There was also an incidental finding of a neuroendocrine tumor grade 1, pT1a, in the appendix.

The patient suffered from a postoperative infection, but recovered well. No adjuvant chemotherapy was given. She is under follow up and there are no signs of recurrence 9 months (Fig. 1d) after surgery.

## Discussion

There have been several studies with conflicting results regarding the prognosis of patients with MAC compared to NMAC [2, 3]. A meta-analysis from 2011, with 44 studies and 222 256 patients with CRC included, stated that mucinous differentiation resulted in an increased hazard of death [2]. It has been suggested that a worse prognosis could be due to the fact that MAC more often present at a more advanced stage [12], but the meta-analysis from 2011 showed that a worse prognosis persisted after correction for stage [2].

On the other hand, in 2012 a study of 244 794 patients with CRC showed that there was no difference between the groups when MAC was located in the colon. The exception was signet-ring cell adenocarcinoma, which had a significantly worse prognosis, regardless of stage and location.

A disadvantage when comparing studies including patients with MAC is that even though WHO has defined MAC, several studies have used their own definition, often with a higher percentage of mucinous component, and no distinction between MAC and signet-ring cell carcinoma [2]. Due to the lack of a precise definition of MAC, it is difficult to interpret the data.

However, studies have shown that MAC is less likely to respond to radiotherapy and conventional chemotherapy [10, 11]. This may be due to the interference with immunologic recognition of tumor cells by masking antigenic epitopes and inhibiting lymphocytic infiltration [13].

LDM chemotherapy seems likely to work through completely different mechanisms than conventional chemotherapy. The mechanisms of LDM chemotherapy are believed to be primarily related to inhibition of tumor angiogenesis [14], which is also the target for vascular endothelial growth factor inhibitors such as bevacizumab. The anti-angiogenic effect of LDM chemotherapy is believed to be due to either direct killing or inhibiting of endothelial cells in the tumor vasculature, or killing of bone-marrow-derived endothelial progenitor cells. Continuous administration of LDM chemotherapy prevents endothelial cells from recovering, which leads to sustained anti-angiogenic effect. In addition, studies have shown that LDM chemotherapy stimulates the immune system through inhibition of T_reg_ cells, which leads to increased activation of both tumor specific and tumor un-specific effector cells. LDM also activates dendritic cells, thereby further inducing the immune-stimulatory effect.

It has been shown that LDM chemotherapy, besides directly affecting tumor cells, also enhances the effect of targeting drugs. Most clinical trials have shown that LDM chemotherapy is well tolerated; high grade toxicity is rare or not found [14]. Many phase II studies have shown the clinical benefit of LDM chemotherapy, including promising tumor control rates and an excellent safety profile [15].

The concept of LDM capecitabine has been successfully used predominately in breast cancer [16] but also in metastatic CRC [17, 18]. Miger et al. [17] retrospectively studied 35 patients with stage IV gastrointestinal cancer that had been treated with LDM capecitabine. Twenty-two of the patients had CRC. The treatment was well-tolerated and 40 % of the patients with CRC had stable disease after 2 months. Two of the patients with CRC lived more than 24 months after initiating treatment. These two patients both had MAC. Romiti et al. [18] retrospectively studied 68 patients with recurrent CRC that had been treated with LDM capecitabine. They concluded that the treatment was well-tolerated and that 19 % of the patients were progression free for more than 6 months. The median overall survival was 23 months for responders.

According to a recently published review, there have been several studies analyzing the effect of the combination of LDM chemotherapy and bevacizumab in patients with glioblastoma multiforme, metastatic ovarian cancer, advanced breast cancer and non-small cell lung cancer [19]. These studies have demonstrated clinical evidence of anti-tumor efficacy, although interpretation of the studies is generally limited by small sample sizes and lack of a control arm. To our knowledge there is only one study which has investigated the effect of LDM chemotherapy and bevacizumab in patients with metastatic colorectal cancer. This study, by Kelley et al., included 35 patients that had received at least two previous lines of therapy, before introducing the combination of cyclophosphamide 50 mg daily with imatinib 400 mg daily and bevacizumab 5 mg/kg every 2 weeks. The treatment was well tolerated and led to prolonged (more than 6 months) stabilization of the disease in 20 % of patients [20].

The current patient had a series of unfavorable prognostic factors besides having a mucinous adenocarcinoma: rapidly growing locally advanced tumor, thrombocytosis [21] and KRAS mutation [22]. Conventional therapy caused severe infections and a pulmonary embolism and the dose was reduced (Fig. 3, Table 1). The result was disappointing, since the tumor size was unaffected. Even though the treatment was now focused on palliation, the patient was young and highly motivated to receiving treatment. The only thing deemed feasible was LDM capecitabine, which has been used in previous studies in our clinic with promising results [17, 23]. The patient was followed up frequently and when the treatment was well tolerated, it was combined with bevacizumab. After 4 months, a reduction in tumor size was seen and the tumor center was low attenuating according to the CT. Postoperatively the center of the tumor was confirmed to be necrotic. Nine months after surgery she was still recurrence free. To our knowledge this is the only case where the cancer in a patient with multifocal metastatic MAC with colorectal origin has been successfully and completely eradicated.

## Conclusion

LDM capecitabine in combination with bevacizumab could be an attractive therapy option for treating patients who are not eligible for more advanced treatment. As the present case exemplifies, in addition to being feasible, it could give a remarkable result. However, the described case report highlights the need for future studies to evaluate the outcome of this treatment.

## Consent

Written informed consent has been obtained from the patient for publication of this case report and accompanying images. A copy of the written consent is available for review by the Editor-in-Chief of this journal.

## Abbreviations

CA 19–9: cancer antigen 19–9
CEA: carcinoembryonic antigen
CRC: colorectal cancer
CT: computed tomography
FLOX: fluorouracil, folinic acid and oxaliplatin
LDM: low dose metronomic
MAC: mucinous adenocarcinoma
MDC: multidisciplinary conference
NMAC: non-mucinous adenocarcinoma
